# Role of ginsenosides in reactive oxygen species-mediated anticancer therapy

**DOI:** 10.18632/oncotarget.23407

**Published:** 2017-12-19

**Authors:** Islam M.D. Sodrul, Chenying Wang, Xiangfeng Chen, Jing Du, Hongxiang Sun

**Affiliations:** ^1^ Key Laboratory of Animal Virology of Ministry of Agriculture, College of Animal Sciences, Zhejiang University, Hangzhou, China; ^2^ Departments of Physiology and Pharmacology, Bangabandhu Sheikh Mujibur Rahman Agricultural University, Gazipur, Bangladesh

**Keywords:** ginsenosides, ROS, cancer, anticancer therapy, mechanism

## Abstract

Cancer is still a global public health problem, which is the leading cause of death in most countries. Ginseng has been used for centuries all over the world as a panacea that promotes longevity. As the king of herb plants, ginseng holds great promise as a new treatment option which is used either by itself or in combination with other medicinal ingredients that is widely accepted as complementary and alternative medicine in cancer therapy. Ginsenosides, the major pharmacologically active ingredients of ginseng, have been shown to have multiple medicinal effects including prominent anticancer activity. The purpose of this review is to give our perspective about the roles of ginsenosides in reactive oxygen species (ROS)-mediated anticancer therapy. Additionally, to provide new sheds light for further improvement and carry out pre-clinical and clinical trials to develop it successfully into a potential anticancer agent. *Panax* herbs and their derivate/metabolites ginsenosides exert beneficial effects for treating various types of cancers. The mechanism of ROS-mediated anticancer activities of ginsenosides varies depending on the specific type of cancer cells involved. Ginsenosides may suppress cancer cell proliferation through anti-oxidation on tumor initiation and induce apoptosis, paraptosis or autophagy *via* generation of ROS on tumor progression, promotion, angiogenesis, invasion and metastasis by various signaling pathways e.g., activation of AMPK, MEK, ASK-1/JNK, ESR2-NCF1-ROS, ER-dependent PI3K/Akt/Nrf2, P53-CHOP, ROS-JNK-autophagy, and/or inhibition of PI3K/Akt signaling pathways. These multiple effects rather than a single may play a crucial role in emerging ginsenosides as a successful anticancer drug.

## INTRODUCTION

Cancer is a multifactor disease and it represents leading causes of death for both men and women all over the world [1], with approximately more than 18 billion cancer deaths projected to occur in the next five years [2]. In recent decades, an increase in mortality and morbidity rates of cancer has been demonstrated in developed or developing countries because of different types of pollutions (especially environmental), carcinogenic agents, unhealthy lifestyles, anxiety, and heavy workload [1]. Presently, quick changing cancer prevalence occurs throughout the world. National Cancer Institute of USA projected that, a total of 1,688,780 new cancer cases, as well as 600,920 cancer related deaths will be occur in 2017 in USA (National Cancer Institute SEER Data. [(Accessed on 25 June 2017)]; Available online: http://seer.cancer.gov/statfacts/).

Despite the significant development made in diagnosis and treatment, the current clinical therapies including radiotherapy, phototherapy, chemotherapy, immunotherapy and surgery are limited as indicated by the salient morbidity and mortality rate as well as low outcome from cancer, suggesting crucial demands for novel efficient therapeutic agents [2, 3]. Current treatment options mainly based on chemotherapy that significantly reduce therapeutic success in cancer owing to the presence of several disadvantages like multiple forms of drug resistance, severe adverse effects and complications, such as fatigue, pain, diarrhea, nausea, vomiting, and hair loss [1, 4]. Therefore, much attention has been paid for exploring a novel drug from natural sources with few side effects that is highly effective for cancer treatment is of great importance.

Recently, about 80% commercially available anticancer new drugs derived from natural resources such as from marine organisms, micro-organism and plants sources [5]. However, the less success rates of chemotherapies, multiple forms of drug resistance, severe adverse effects and complicacy emphasize the importance of discovering new compounds, which is considered as safe, cost effective and alternative to cancer treatment [6–8].

Complementary and alternative medicine (CAM), also known as “other than” conventional medicine, is a group of diverse medical and healthcare systems, practices and products [3]. Globally, approximately 9.8–76.0% of the populations have used some form of CAM that greatly varies from country to country [9]. Among the different kinds of CAM, ginseng has been extensively used in the oriental and presently in western medicines whose history stretches back nearly 5,000 years [10]. Etymologically, *Panax* means “all-healing” in Greek and people's belief that it is a panacea that promotes longevity [11]. The name “*ginseng*” came from a Chinese word “rénshen” which literally means “essence of men” [4]. Genus *Panax* belongs to the family Araliaceae, which comprises of 11 different species, including *P. ginseng* C.A. Meyer (known as Asian or Korean ginseng), *P. quinquefolius* L. (American ginseng), *P. notoginseng* (Burkill) F.H. Chen (Chinese ginseng, also called Sanchi or notoginseng), are vital herbs that used to treat different medical conditions [12].

Pharmacological actions of ginseng are mainly imputed to a variety of ginsenosides, a triterpenoid saponins that are secondary metabolites uniquely present in *Panax* species. Other than saponins, ginseng also contains flavonoids, polyacetylenes, phytosterols, essential oils, acids, polysaccharides, nitrogen-containing compounds and vitamins [10, 13]. The ginseng root contains 2–3% ginsenosides and also found in other parts of this herb such as leaves, flowers and berries [14]. More than 180 known different ginsenosides with various numbers, linkage positions and types of sugar moiety have been isolated and identified since their first discovered in the 1960s by Shibata's group [15, 16]. Amazingly, the antitumor activities of ginsenosides negatively linked to the number of sugar groups, the number and position of hydroxyl groups and stereoselectivity [17].

**Figure 1:**
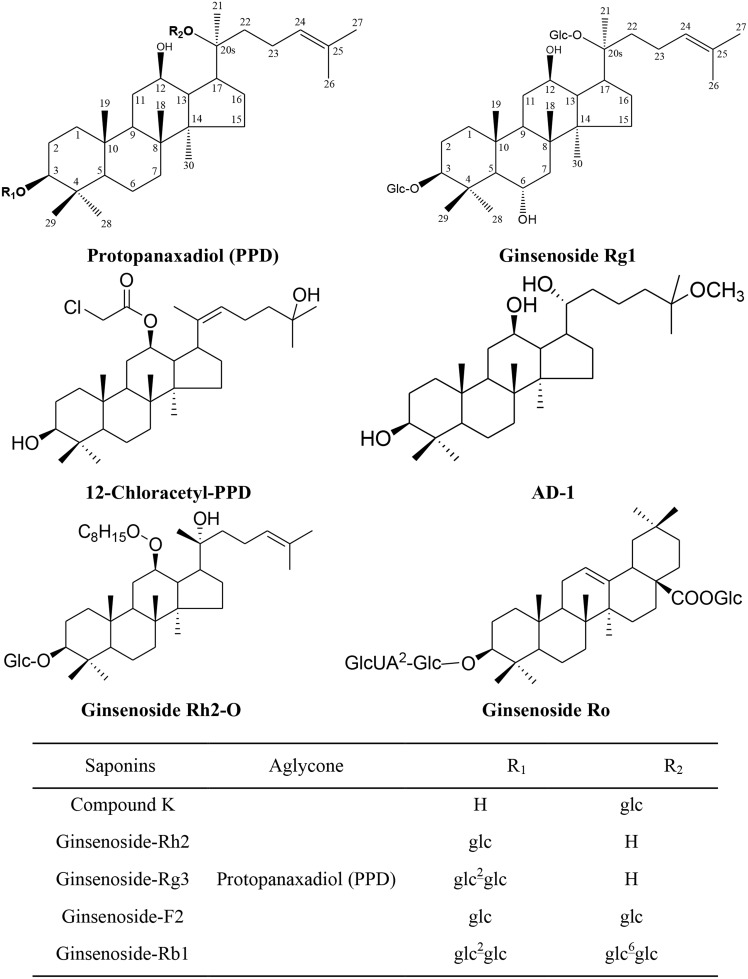
Chemical Structure of Ginsenosides Included in This Review Glc: *β*-D-glucopyranosyl; GlcUA: *β*-D-glucuronyl-.

Ginsenosides can be divided into two major types based on the functional groups at C6 position, namely (1) protopanaxadiol type (PPD) (*e.g.*, ginsenoside Rb1, Rb2, Rc, Rd, Rg3 and Rh2) contains a hydrogen atom at C6, (2) protopanaxatriol type (PPT) (*e.g.*, ginsenoside Re, Rf, Rg1 and Rh1) contains a C-6 sugar chain. The minor types are oleanolic acid type (e.g., ginsenoside Ro) and ocotillol type (*e.g.*, pseudoginsenoside) (Figure 1). Protopanaxadiol ginsenosides exhibits higher anticancer activities than protopanaxatriol type. Moreover, the anticancer ability of ginsenosides depends on the position of sugar linkages in the order: C-3 > C-6 > C-20 [18]. American ginseng manifested a greater affinity for free radicals scavenging and capable of delaying lipid peroxidation as well as higher content of ginsenosides compared to Asian ginseng [19, 20].

**Figure 2:**
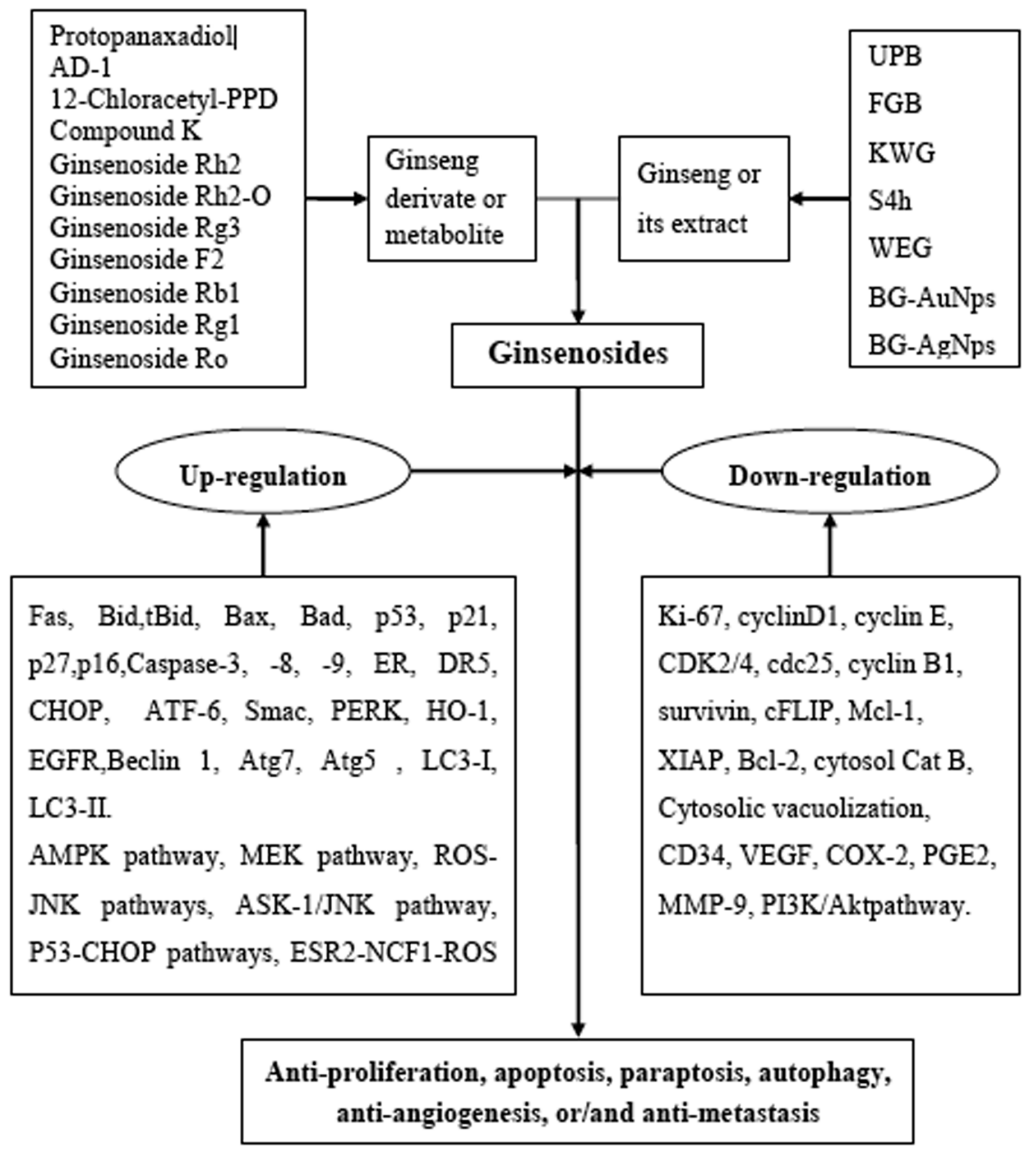
Schematic Diagram of Reactive Oxygen Species (ROS)-Related Anticancer Effects Mediated by Ginsenosides Up-regulation: Fas, Bid, tBid, Bax, Bad, p53, p21, p27, p16, caspase-3, caspase-8, caspase-9, ER (endoplasmic reticulum) stress, DR5, CHOP, ATF-6, Smac, PERK, HO-1, EGFR, Beclin 1, Atg7, Atg5, LC3-I, LC3-II, AMP-activated protein kinase (AMPK), MEK signaling pathway, ASK-1/JNK signaling pathway, estrogen receptor 2 (ESR2)-neutrophil cytosolic factor 1 (NCF1)-ROS signaling pathway, ER (estrogen receptor)-dependent PI3K/Akt/ Nrf2 pathway, P53-CHOP pathways, and ROS–JNK–autophagy pathways. Down-regulation: Ki-67, cyclinD1, cyclin E, CDK2/4, cdc25, cyclin B1, survivin, cFLIP, Mcl-1, XIAP, Bcl-2, cytosol Cat B, cytosolic vacuolization, CD34, VEGF, COX-2, PGE2, MMP-9 and PI3K/Akt signaling pathway.

The scavenging effects of ginsenosides on intracellular ROS were ranked in the decreasing order: Rc > Rb2 > Rg2 > Rh2 > Rh1 > Rf > Rg3 > Rg1 > Rb1 > Re > Rd [21]. Recently, various reports have demonstrated that ginsenosides may be capable of enhancing therapeutic potential of conventional chemotherapeutic agents in case of cancer patients through defending normal tissues from chemotherapy-induced damage [4].

Ginsenosides and ginseng extract have been proved to possess an antioxidant, anti-inflammation, anti-fatigue, anti-stress, cure for respiratory problems, hemocytopenia therapy, cardioprotection, anti-obesity, anti-diabetic, immunomodulation, neuroprotection, antimicrobial action, sexual potentiating, antitumor [22], anti-allergic [23], antihypertensive [24] activities. Ginsenosides have been postulated to exert anticancer activities through cellular and molecular targets via modulation of diverse signaling pathways, thereby inhibiting the tumor by regulation of cell proliferation, inhibition of oxidative damage and inflammation, induction of apoptosis and autophagy, preventing angiogenesis, delaying invasion and metastasis, regulating tumor-related immune suppression, and increasing the sensitivity of resistant cells to chemotherapeutic agents [4, 17, 25].

Ginseng is popularly used in cancer patients, and several studies have assorted with ginseng consumption and risk of cancer prevention and treatment [4]. Various case-control studies proved that clinical uses of ginsengs have cancer prevention ability in the oral cavity, stomach, lung, liver, pancreas, ovary and colon by regular consumption [26]. Yun and Choi conducted a prospective cohort study to evaluate the preventive effect of ginseng against cancer on a population residing in a ginseng cultivation area on the basis of the result of case-control studies, and drew the conclusions that *P. ginseng* has non-organ specific preventive effect against cancer [27]. A 5 years follow-up cohort study of about 4600 non-cancer individuals revealed that risk of cancer considerably lower among ginseng intakers than non-intakers [28]. In a large, population-based cohort study of 1455 breast cancer patients in China showed that regular consumption of ginseng at a dose of 1.3 g per day before cancer diagnosis and nonstop to use it after the diagnosis had significantly decreased risk of death and recurrence equated with patients who never used ginseng [29]. Regular use of ginseng after cancer diagnosis was also positively linked with the quality of life scores, significant result in the psychological and social well-being domains [29]. In other study involving a meta-analysis of 274 female breast cancer patients in China also found that the combining treatment group showed considerably attenuated leucopenia and progress Karnosfsky Performance Scale score after chemotherapy [30].

It was reported that red ginseng powder inhibited the recurrence of AJCC stage III gastric cancer and showed immunomodulatory activities during postoperative chemotherapy, after a curative resection with D2 lymph node dissection [31]. In a randomized, double-blind, placebo-controlled pilot trial of 53 cancer patients, sun ginseng (3000 mg/day) was given daily for 12 weeks resulting in helpful ameliorating some expressions of mental and physical functioning, in case of gynecologic cancer and hepatobiliary cancer patients [32]. Another randomized, double-blind clinical study of 290 cancer patients also revealed that regular consumption of 750 mg of *P. quinquefolius* (American ginseng) does not contribute any benefit compared to the placebo, but two high doses (1000–2000 mg/day) did seem to reduce cancer-related fatigue more than a placebo [33]. In a recent randomized, double-blinded, placebo-controlled trial, 643 patients with chronic atrophic gastritis in China was given red ginseng extract powder (1 g/week) for 3 years and carried out for 8 years, and found that the administration of red ginseng extract powder for 3 years exerted significant preventive effects on the incidence of non-organ-specific human cancers in males [34].

A recent meta-analysis involving 7,436 cancer patients and 334,544 participants showed that ginseng consumption is associated with a significantly decreased risk of cancer (16%) and that the effect is not organ specific [35]. In a more recent randomized, double-blinded, placebo-controlled trial, 30 patients with epithelial ovarian cancer in Korea were given placebo or red ginseng (3000 mg/day) for three months. The results suggested that red ginseng may be capable of decreasing genotoxicity and enhancing health-related quality of life despite no benefit of survival in patients with epithelial ovarian cancer who received chemotherapy [36].

However, some contradictory and puzzling results were also come forward. For example, a large case–control study and a small cohort study have together proposed that ginseng consumption is related with a 60–70% decrease risk of gastric cancer in Korean populations. However, when the study was tried to be repeated in a large prospective cohort study of Chinese women, no relationship was found between ginseng consumption and risk of gastric cancer patients [37]. A retrospective case-control study of 949 breast cancer patients also showed that there is no significant relationship between ginseng consumption and risk of breast cancer patients [38]. The U.S. Food and Drug Administration (FDA) rates ginseng as GRAS (generally recognized as safe), which is generally known as a CAM in treatments of different types and stage of cancer in the U.S. and Europe [4].

In the past 30 years, massive attempts have also been made to recognize the anti-cancer activity of ginseng compounds, such as ginsenoside Rg3 and Rh2. Among them, some compounds are currently accessible as over-the-counter drugs in China and universal. Shenyi capsule (ginsenoside Rg3 monomer preparation), an autonomously formulated class A new anti-cancer drug in traditional Chinese medicine, has been generally applied clinically in the treatment of different types of cancer, including breast cancer, gastrointestinal tumors and lung cancer in China [39]. A prospective, randomized, controlled study of 133 non-small cell lung cancer patients exhibited that Shenyi capsule, mainly in combination with chemotherapy, augmented the post-operative lifespan of patients was also positively linked with increasing the immune function and suppressing angiogenesis [40]. Another randomized trial on 60 patients with advanced esophageal cancer patients establish that Shenyi capsule in combination with chemotherapy was efficient in ameliorating the patients’ quality of life and 1 year survival rates [41].

In addition, some preparations, mainly consisted of ginseng, were evaluated for the effects on the human cancer. One clinical and experimental study on 176 cases of digestive tract cancer revealed that Shenqi injection enhanced immune function and body weight of the patients and decreased toxic effects of chemotherapy [42]. Another randomized trial of 63 stomach cancer patients showed that Shenmai injection augmented human immune function after chemotherapy [43].

To the best of our knowledge, this is the first systemic review about the roles of reactive oxygen species (ROS) in anticancer therapy with ginsenosides (Table 1). Based on the evidence manifesting anticancer properties of ginsenosides and the roles of ROS in cancer biology, this review summarizes recent advances about the ROS-mediated anticancer effects of ginsenosides and fetches new sheds light for further design of anticancer research or more conduct of preclinical and clinical trials with this king of herb plants.

**Table 1:** *Panax* herbs and their derivate or metabolites compounds of ginsenosides that modify ROS-related effects on cancer cells^*^

Components [Ref]	Cancer cells	Concentrations		Molecular targets	Signaling pathway	Effects
		Conc. range	IC_50_			
Ginsenoside Rh2 and Rg3 [44]	Leukemia Jurkat cells	0-60 μM	35 μM (24 h)	ROS↑, MTP↓, caspase-3/9↑, Bax/Bcl-2↑, Cyt C↑	Mitochondria-dependent apoptotic pathway	Inhibits cell proliferation and induces apoptosis by stimulating the mitochondrial ROS generation
20(S)-Ginsenoside Rh2 [45]	Acute lymphoblastic leukemia cells	0-60 μM	35 μM (24 h)	ROS↑, MTP↓, Cyt C↑, caspase-3/9↑, LC3-I↑, LC3-II↑, Atg5↓, Beclin-1↑	Mitochondria-dependent apoptotic pathway	Inhibits autophagy and induces apoptosis by mitochondrial ROS generation
	Reh cells		40 μM (24 h)			
Ginsenoside Rg3 [46]	Lewis lung	0-600 ng/ml	NA	CDK2↓, cyclin E↓, CDK 4↓, cyclinD1↓, ERK↓, p38↓, JNK↓, BAX↑, PARP↑, Bcl-2↓	Cell proliferation-associated pathways	Induces apoptosis by inhibiting activation of MAPK through the regulation of intracellular ROS.
AD-1 [47]	lung cancer A549 and H292 cells	0-50 μM	6.47 μM (A549)	p38↑, ERK1/2↑, VEGF↓, MMP-9↓, CD34↓, MDM2↓, cyclin D1↓, cyclin E↓, p21↑, p27↑, Bax↑, Bcl-2↓, G0/G1↑	p38 MAPK pathway	Inhibits cell proliferation and induces apoptosis through ROS generation and p38 MAPK activation
			3.46 μM (H292)			
Compound K [48]	Lung cancer NCI-H460 cell	30 μg/mL	NA	ROS↑, MMP↓, caspase-3↑	Intrinsic apoptotic pathways	Enhances γ-ray induced apoptosis by increasing intracellular ROS generation, loss of MMP and activation of caspase-3
Ginsenoside Rh2 [49]	Colorectal cancer HCT-116	0.5-50 μM	2.5 μM/24 h (Sodium selenite)	ROS↓, Bax/Bcl-2↑, capase-3↑, G1↓, S ↓	Intrinsic apoptotic pathways	Induces anti-proliferative activity and autophagy by depletion of ROS production
			12.5 μM (24 h) (G-Rh2)			
Ginsenoside Rh2 [50]	Colorectal carcinoma HCT-116 and SW-480 cells	0-60 μM	35 μM (72 h)	Bax↑, Bad↑, Bcl-2↓, Bcl-XL↓, ROS↑, NF-κB↑, p53↓, Cytosolic vacuolization↓	p53 and NF-κB signaling pathway / ROS-NF-kB pathway	Induces apoptosis and paraptosis by activating both p53 and NF-kB through up-regulation the levels of ROS
Protopanaxadiol [51]	Colorectal cancer HCT-116 and SW-480cells	10–50 μM	35 μM (24 h)	ROS↑, NF-κB↑	NF-κB pathway	Induces paraptosis through activation of the NF-κBpathway by ROS generation
S4h [52]	Colorectal cancer HCT-116 and SW-480 cells	0.2 mg/ml (HCT-116)	NA	ROS↑, MTP↓, Bcl-xL↑, NF-κB↑, IκB↓	Both the apoptosis pathway and the ROS/NF-κB mediated survival pathway	Induces apoptosis by activating both the apoptosis pathway and the ROS/NF-kB mediated survival pathway.
		0.4 mg/ml (SW-480)				
Compound K [53]	Colon cancer HT-29 cells	10-40 μg/mL	20 μg/mL	MTP↓, caspase-3/9↑, Cyt C↑, Bax↑, Bcl-2↓, ERK↓, JNK↑, p38↑	Mitochondria-dependent apoptotic pathway	Induces apoptosis by generation of ROS via mitochondria-dependent apoptotic pathway and MAPK pathway
Compound K [54]	Colon cancer HT-29 cells	NA	20 μg/ml (24h)	PERK↑, eIF2α↑, Ca2+↑, ER stress↑, IRE-1↑, XBP-1↑, ATF-6↑, GRP-78↑, CHOP↑, caspase-12↑	Endoplasmic reticulum (ER) stress signaling pathway	Induces apoptosis which is mediated by ER stress signaling pathway
Compound K [55]	Colon cancer HCT-116 cells	0-50 μg/ml	20 μg/ml (24h)	Caspase-3/9↑, GFP-LC3↑, LC3-I↑, LC3-II↑, Bcl-2↓, Bax↑, Atg5↑, Atg6↑, Atg7↑, JNK1/2↑	JNK pathway	Induces autophagy and apoptosis through ROS generation and JNK activation
Compound K [56]	Colon cancer HCT-116 cells	0-50 μM	NA	ROS↑, Mcl-1↓, Bcl-2↓, survivin↓, XIAP↓, cFLIP↓, Bax↑, tBid↑, Cyt C↑, LC3-II↑, Atg7↑, JNK↑, ERK↓, p38↓, p53↑, DR5↑, CHOP↑	p53-CHOP and ROS–JNK–autophagy pathways	Enhance TRAIL-induced apoptosis through autophagy-dependent and–independentDR5 up-regulation.
Ginsenoside Rg3 [57]	Colon carcinoma CT-26 cells	10-50 μM	NA	Ki-67↓, VEGF↓, CD34↓, HO-1↑, NQO-1↑, Nrf2↑	Nrf2-mediated HO-1/NQO-1	Promotes the efficacy of cisplatin via preventing cisplatin-induced intracellular ROS generation.
Korean white ginseng extract [58]	Hepatoma HepG2 Cells	18.6 μg/mL	NA	Cyt C↑, c- Jun↑, SAPK↑, MDA↓, caspases-3↑, Iκ-b↓	JNK–NF- κB –cytochrome c apoptotic pathway	Induces apoptosis *via* an antioxidative effect and JNK–NF-κB–Cyt C apoptotic pathway
Fermented black ginseng [59]	Hepatoma HepG2 Cells	10-200 μg/ml	50 μg/ml	ROS↓, Bax/Bcl-2↑, capase-3↑, G1↓, S ↓	Intrinsic apoptotic pathways	Induces anti-proliferative activity and autophagy by depletion of ROS production
UGB [60]	Hepatoma HepG2 Cells	5-75 μg/ml	20 μg/ml (24h)	ROS↑, caspase-3↑, Bax↑, Bcl-2↓	Intrinsic apoptosis pathway	Induces apoptosis through an intrinsic apoptosis pathway
Ginsenoside Rh2 [61]	Hepatoma HepG2 Cells	5-55 μM	42.12 μM (Rh2)	Caspase-3/9↑, Bcl-2↓, Bax↑, PARP↑, tBid↑, Cyt C↑, MMP↓	Mitochondrial-mediated intrinsic pathway	Induces apoptosis through a mitochondrial mediated intrinsic pathway via generation of intracellular ROS
			20.15 μM (Rh2-O)			
Ginsenoside Rh2 [62]	Hepatoma HepG2 Cells	0-50 μM	42.12 μM (24h)	Caspase-3/9↑, cytosol Cat B↓, leupeptin (Leu) ↑, MTP↓, Bid↑, tBid↑, Cyt C↓	Lysosomal-mitochondrial apoptotic pathway	Induces apoptosis through ROS accumulation and mitochondrial apoptotic pathway
Ginsenoside Rh2 [63]	Hepatoma HepG2 Cells	25-50 μM	NA	PARP↑, ROS↑, p-p38↑, p-AMPK↑	AMPK signaling pathway	Induces apoptosis through activation of AMPK-mediated ROS generation.
Ginsenoside Rg3 and Rh2 [64]	Hepatoma Hep3B cells	3-50 μM	1-30 μM (24 h)	ROS↑, caspase-3↑, Bcl-2↓, Bax↑, Cyt C↑, MTP↓	Mitochondria-mediated apoptosis pathway	Induces apoptosis through intracellular ROS production and mitochondria-mediated apoptosis pathway
Ginsenoside Rg3 [65]	Hepatocellular carcinoma cells	100 μmol/L	12.5 ng/mL (TRAIL)	Caspase-3↑, PARP↑, eIF2α↑, CHOP↑, GRP78↑, DR5↑	NA	Induces sensitization of TRAIL-induced apoptosis via CHOP-mediated DR5 up-regulation.
Compound K [66]	Breast cancer MCF-7 cells	10-50 μg/ml	35 μg/ml (48 h)	MTP↓, AMPK↑, COX-2↓, PGE2↓	AMPK–COX-2 pathway	Induces apoptosis via generation of ROS and modulation of AMPK signaling
Ginsenoside Rg3 [67]	Breast Cancer MDA-MB-231 Cells	0-50 μM	30 μM (24 h)	ROS↑, Bax /Bcl-2↓, MTP↓, caspase-3↑, PARP↑	Mitochondrial death pathway	Induces apoptosis by the activation of the mitochondrial death pathway.
Ginsenoside Rg3 [68]	Breast Cancer MDA-MB-231 Cells	10-30 μM	30 μM (24 h)	mutant p53↓, p-ERK↓, Akt↓, NF-κB↓, Bcl-2↓, p65↓, p53↑, IκBα↓, MDM2↑, IKKβ↓,	NF-κB pathway	Inhibits NF-κB signaling via inactivation of ERK and Akt as well as destabilization of mutant p53.
BG-AuNps and BG-AgNps [69]	Breast cancer MCF-7 cells	1-100 μg/mL	3 μg/mL (BG-AuNps)	ROS↑	NA	Induced oxidative cell damage and apoptosis through ROS generation.
			2.05 μg/mL (BG-AgNps)			
12-Chloracetyl-PPD [70]	Prostate cancer C4-2B cells	0-30 μM	9.85± 0.62 μM	ROS↑, MDM2↓, p53↑, p21↑, cdc2↓, cdc25C↓, cyclin B1↓, G1 phase↓, G2/M phase↑ cdc2-Tyr15↑, cdc25-Ser216↑, caspase-3/8/9↑, PARP↑	G2/M cell cycle arrest	Inhibits proliferation and induces ROS-mediated cell apoptosis *via* down-regulated MDM2 expression and up-regulated p53 expression.
Ginsenoside Rh2 [71]	Cervical carcinoma HeLa cells	7.5 μg/mL	NA	ROS↑, MTP↓, Caspase-3↓, JNK1↑, SEK1↑, JNK2↑, c-Jun↑, Smac↑, Bax↑, Ca^2+^↑	ROS-JNK1 pathway	Induces apoptosis by Ca^2+^ and ROS generation leading to the activation of SEK1 and JNK1
Compound K [72]	Bladder cancer T24 cells	0–25 μM	5 μM (4 h)	Cyt C↑, Bax↑, Bcl-2↓, p-p38MAPK↑, procaspase-3/9↑, p38↑, ROS↑, glutathione↓	p38MAPK pathway	Induces apoptosis via ROS-mediated p38 MAPK pathway
Ginsenoside F2 [73]	Gastric carcinoma SGC7901 cells	10-40 μg/mL	20 μg/mL	ROS↑, MTP↓, PARP↓, ASK-1↑, JNK↑, Bcl-2↓, Cyt C↑, Caspase-3/9↑	ASK-1/JNK pathway	Induces apoptosis through ROS-mitochondria pathway and modulation of ASK-1/JNK signaling cascade
GinsenosideRo [74]	Esophageal cancer cells	25-200 μM	NA	ROS↑, CYBB/Nox2↑, LC3B-II↑, ATG7↑, ESR2↓, NCF1↑, SQSTM1/p62↑, CSTB↓ CSTD↓, p-CHEK1↑, EGFR↑, DDIT3↑, ATM↑, ATR↑, BRCA1↑, GFP-LC3B puncta↑, Lysosomal pH↑, autophagic vacuoles↑	ESR2-NCF1-ROS pathway	Suppresses autophagy via the ESR2-NCF1-ROS signal pathway and sensitized 5-fluorouracil-induced cell death through reducing CHEK1 degradation.
Ginsenoside Rh2 [75]	Rat C6 gliomalcells	7.5 -10 μg/mL	7.5 μg/mL (6 h)	PARP↓, Bcl-XL↑	Caspase pathway	Accelerates apoptosis is mediated by the generation of ROS, DNA fragmentation and the initiation of caspase pathway in a Bcl-XL-independent manner
Ginsenoside Rg1 [76]	Neuroblastoma SH-SY5Y cell	5-20 μM	10 μM	ROS↓, JNK↑, caspase-3↑	JNK and caspase-3 pathway	Prevents MPP^+^-induced apoptosis by inhibiting production of ROS and activating JNK pathway
Compound K, Ginsenoside Rh2 [77]	Astrocytoma CRT-MG cells	0~50 mg/L	25 mg/L (6 h)	ROS↑, caspase-3↑, Cyt C↑, p-p38↑, Fas↑, MIP↓	Distinct apoptotic pathways	Enhance Fas-mediated apoptosis in a caspase-, mitochondria-, and ROS-dependent manner.
Ginsenoside Rb1 [78]	Neuroblastoma SH-SY5Y cell	10–100 μg/ml	NA	HO-1↑, caspase-3/9↓, PI3K↑, ROS↓, Akt↓, Nrf2↑	Gβ1/PI3K/Akt-Nrf2 pathway	Prevents dopamine-induced oxidative stress through estrogen receptor-dependent Gβ1/PI3K/Akt-Nrf2 signaling pathway
WEG [79]	Neuroblastoma SH-SY5Y cell	0.01-0.2 mg/mL	NA	ROS↑, Bcl-2↓, Bax↑, Cyt C↑, caspase-3↑	Mitochondria-dependent apoptotic pathway	Prevents MPP+-induced apoptosis by alleviating oxidative stress and mitochondria-dependent apoptotic pathway.
20(S)-ginsenoside Rg3 [80]	GliomaU87cells	10-100 μM	20 μM	ROS↑, p21↑, p16↑, p53↑, Akt↑	Akt and p53/p21-dependent pathways	Induces senescence-like growth arrest by increasing ROS generation via Akt and p53/p21-dependent pathways
Ginsenoside Rg3 [81]	Glioblastoma U87MG cells	10 μM	NA	Bcl-2↓, Bax↑, pro-caspase3↓, MEK1/2↑, ROS↑	MEK pathway	Induces apoptosis through the MEK signaling pathway and ROS

^*^S4h, 4 h-steamed American ginseng root extract; UGB, ultrasonication processed *Panax ginseng* berry extract; WEG, water extract of ginseng; Cyt C, cytochrome C; MIP, mitochondrial intermembrane potential; MPP+, 1-methyl-4-phenylpyridinium ion; MTP, mitochondrial transmembrane potentials; ↑, up-regulation; ↓, down-regulation,; NA, Not available; IC_50_, half maximal inhibitory concentration.

## MATERIALS AND METHODS

We conducted a literature search for relevant articles in PubMed, Ovid Technologies, Excerpta Medica (EMBASE), Scopus databases, Google scholar, pubmed.cn, Web of Science SCI, Cochrane Library, Chinese National Knowledge Infrastructure (CNKI), and for published papers until May 2017 using the following search terms: “reactive oxygen species and cancer and Ginsenoside OR Ginseng” or “reactive oxygen species and cancer and Ginseng saponin”. Studies were screened for relevance. The contents of the identified articles were summarized and the current review focused on the ROS-mediated anticancer effects of ginsenosides. After removing duplicate publications and excluding information that was unrelated to ROS, we collected 38 articles about the ROS-related anticancer effects of ginsenosides.

## ROLE OF ROS IN CANCER

Reactive oxygen species (ROS) are a group of highly reactive chemicals containing oxygen owing to the presence of their radicals, ions or molecules that have a single unpaired electron in their outermost shell of electrons. More than 20 types of ROS, among them, hydrogen peroxide (H_2_O_2_), superoxide anion radical (O_2_^·−^), and the highly reactive hydroxyl radical (OH^·^) play a crucial role in cancer [82]. Mounting evidence has demonstrated that ROS actively participate in multistep tumorigenesis, which is related to tumor initiation, transformation, progression, promotion, angiogenesis as well as metastasis [83–86]. Oxidative stress occurs when the imbalance between ROS production and elimination, which may be showed by the overproduction of ROS in the cells or the impairment of the antioxidant defense system [87], while abnormally increased ROS production accelerates irreversible damage to cellular macromolecules including proteins, lipids and DNA, contribute to deadly lesions in cell that initiates carcinogenesis [88]. Cancer cells produce increased numbers of ROS compared to their normal cell counterpart owing to the presence of their increased metabolism [89] and their elevation dependence on an antioxidant defense system [90].

The role of ROS in cancer is two-sided. First, ROS can triggers cancer initiation, progression as well as distributing through the activation and repairs of signaling pathways, which influence proliferation of cells, survival, angiogenesis and metastasis [91–93]. Second, the overproduction of ROS in malignant cells can also initiate signaling of cell death, senescence and cell cycle arrest [94, 95]. Mitochondria in malignant cell characteristically have a massive production of ROS and differ structurally and functionally from their normal cell counterpart, while oncogenic ROS enhance cancer generation *via* inducing genomic instability, altering gene expression, and affects in different signaling pathways [96]. In general, low concentrations of ROS are generated from oxygen through mitochondrial electron transport chain and from NADPH oxidases (NOXs) [97] that are involved for many cellular process and signal transduction. At low to moderate levels, ROS may participate in tumor generation through promotes the mutation of genomic DNA and/ or active signaling molecules. At high levels, ROS induce severe cell damage and death, especially at the beginning stage of tumor growth [98].

The increased ROS levels in cancer cells are a results of changes in mitochondrial metabolism [96, 99] that trouble cellular signaling pathways [100, 101], which are mainly mediated by the transcription factors NF-κB and STAT3, hypoxia-inducible factor-1α, kinases, growth factors, cytokines, and other enzymes [90] associated with cellular transformation, inflammation, tumor cell survival, proliferation, invasion, angiogenesis as well as metastasis of cancer [90].

ROS induce transformation of non-malignant to malignant cells through cellular DNA damage and DNA methylation [102], leading ultimately to mutations that impair oxidative phosphorylation process [96]. Some cancer cells abnormally express ROS-removing antioxidant enzymes and ROS-producing NADPH oxidases. Moreover, increasing evidence has suggested that cancer cell can produce massive ROS that triggers cancer cell death through autophagy [103, 104] and/or apoptosis [101, 105]. Owing to great amount of ROS production cancer cells showed more sensitivity than their normal counterpart [98]. Thus, increasing oxidative stress by exogenous ROS generation could be an efficient approach for the specific killing of cancer cells without causing significant toxicities to normal cells [106].

## ROS-MEDIATED ANTICANCER EFFECTS OF GINSENOSIDES ON VARIOUS CANCER CELLS

*Panax* herbs and their derivates or metabolite compounds including ultrasonication processed *P. ginseng* berry extract (UPB), fermented black ginseng (FGB), Korean white ginseng (KWG), Black ginseng-gold nanoparticles (BG-AuNps) and Black ginseng-silver nanoparticles (BG-AgNps), 4 h-steamed American ginseng root extract (S4h), water extract of ginseng (WEG) contain the active ingredients, aglycone such as protopanaxadiol (PPD), 12-chloracetyl-PPD, AD-1, and glycosides such as ginsenoside Rh2, Rg3, F2, Rb1, Rg1, Ro, and compound K (Figure 1). These compounds have demonstrated anticancer activity (Table 1) with notable dose- and time-dependent inhibitory effects on the viability of leukemia, lung, colorectal, hepatoma, breast, prostate, cervical, esophageal, gastric, bladder, glioma, glioblastoma, neuroblastoma and astrocytoma cancer cells. These effects, in terms of ROS, are described in more detail for each cell type in the following sections.

### ROS-mediated anticancer effects of ginsenosides on leukemia cells

Treatment of human leukemia Jurkat cell with 35μM GRh2 or GRg3 for 24 hours inhibited cell growth in a dose and time-dependent manner. It induces apoptosis by enhancing cleaved-caspase-3 and -9, ratio of Bax to Bcl-2, cytochrome c and mitochondrial ROS generation. Notably, ginsenoside Rh2 was more effective than ginsenoside Rg3 [44]. Xia et al. also reported that 20(S)-ginsenoside Rh2 inhibited human acute lymphoblastic leukemia cells growth in a dose and time-dependent manner with a half maximal inhibitory concentration (IC_50_) of 35μM for 24 hours, and induced mitochondria-dependent apoptosis and autophagy by mitochondrial ROS generation [45].

### ROS-mediated anticancer effects of ginsenosides on lung cancer cells

Ginsenoside Rg3 inhibited the proliferation of Lewis lung carcinoma (LLC) cell *via* suppression of cell cycle-associated proteins, including CDK2, cyclin E, CDK4, and cyclin D1, and cell growth-associated mitogen-activated protein kinases (MAPKs), including extracellular signal-regulated kinases (ERK), p38, and c-Jun NH2-terminal kinase (JNK). The treatment with Rg3 (200 ng/ml) for 48 h induces apoptosis through activation of pro-apoptotic proteins, including BCL-2-associated X protein (BAX), cleaved poly [ADP-ribose] polymerase 1 (PARP1), and cleaved caspase-3 and decreasing of anti-apoptotic proteins, including B-cell lymphoma 2 (BCL-2) *via* the regulation of intracellular ROS level [46].

Another study reported that 20(R)-25-methoxyl-dammarane-3β,12β,20-triol (AD-1) induced G_0_/G_1_ cell cycle arrest in A549 and H292 lung cancer cells followed by decreasing of mouse double minute 2 homolog (MDM2), cyclin D1 and cyclin E, while increasing p21 and p27. Moreover, *in vivo* oral treatment of AD-1 (at a doses of 10-40 mg/kg/day for 6 weeks) dose-dependently suppressed the growth of xenograft tumors by more than 55% (40 mg/kg) without disturbing body weight of athymic nude mice, in association with decreasing the expression of vascular endothelial growth factor (VEGF), CD34, and matrix metalloproteinase-9 (MMP-9) in tumor tissue *via* ROS generation and p38 MAPK activation [47]. In addition, compound K was also reported to enhance γ-ray (10 Gy)-induced apoptosis in NCI-H460 human lung cancer cells *in vitro* by increasing intracellular ROS generation, loss of mitochondrial membrane potential (MMP) and activation of caspase-3. Compound K subcutaneously injected at dose of 30 mg/kg inhibits the growth of xenograft tumors in athymic nude mice *in vivo*. [48].

### ROS-mediated anticancer effects of ginsenosides on colorectal cancer cells

**Figure 3:**
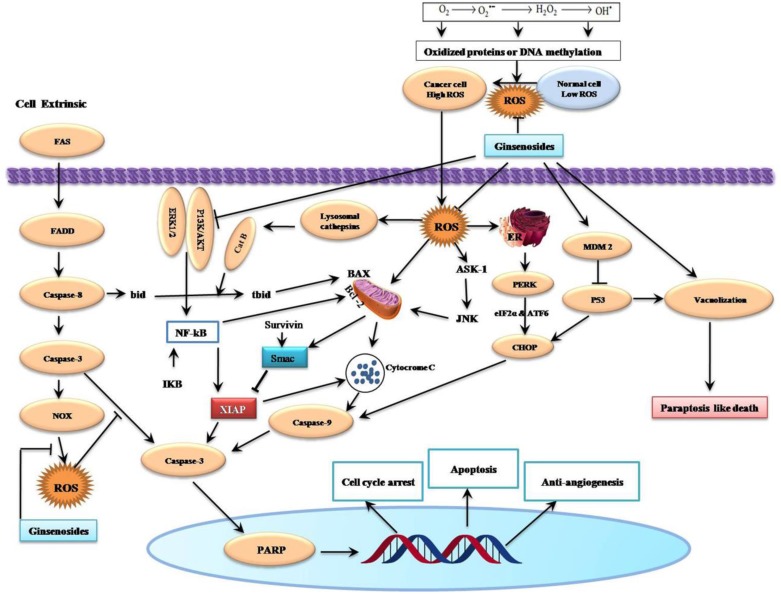
Schematic Diagram of Effects of Ginsenosides on Reactive Oxygen Species-Related Cell Cycle Arrest, Apoptosis, Paraptosis and Anti-angiogenesis of Cancer Cells Ginsenosides can induce intrinsic cell death and they augment Fas-induced extrinsic cell death by ROS suppression. Production of ROS is the vital issues of ginsenosides mediated apoptosis from oxidized protein or DNA methylation. Generally, ROS are generated either through mitochondrial electron transport chain or from NADPH oxidases (NOXs). Aberrant mitochondrial functions are associated with the release of cytochrome c resulting in the activation of caspase-3 and PARP, ultimately leading to apoptosis and cell death. The final effects are cell cycle arrest, induction of apoptosis, paraptosis and anti-angiogenic activity. →, Activation; ┤, Inhibition.

A recent study has shown that the combination of sodium selenite (2.5 μM) and ginsenoside Rh2 (12.5 μM) could induce anti-proliferative activity in a dose- and time-dependent manner. This combination also induce autophagy mediated by cell cycle arrest at the G1 and S phase, elevation of Bax/Bcl-2ratio and capase-3 level and reduction of ROS production in human colorectal carcinoma HCT-116 Cells [49]. Ginsenoside Rh2 induced apoptosis and paraptosis-like cell death in colorectal cancer HCT-116 and SW-480 cells *via* the activation of both the p53 and NF-κB signaling pathway through up-regulation the levels of ROS as well as Bax and Bad, while down-regulation the levels of Bcl-2 (Figure 3) [50].

Protopanaxadiol (PPD) also induced paraptosis in colorectal cancer HCT-116 and SW-480 cells in a concentration-dependent manner through activation of the NF-κB pathway via ROS generation [51]. Li et al. [52] demonstrated that 4h-steamed American ginseng root extract (S4h) accelerated apoptosis in colorectal cancer HCT-116 and SW-480 cells, which is mediated by mitochondria damage that initiates both the apoptosis pathway and the ROS/NF-κB mediated survival pathway.

Compound K inhibited human colon cancer HT-29 cell growth in a dose-dependent manner with IC_50_ values being 20 μg/mL. It significantly induced ROS generation to in turn led to mitochondria-dependent and caspase-dependent apoptosis through regulating the expression of Bcl-2 and Bax, disruption of MMP, release of cytochrome c (Cyt C), elevation of caspase-3, -9, and concomitant PARP cleavage *via* activation of p38 MAPK and JNK pathways [53].

It was also reported that compound K inhibited human colon cancer HT-29 cell growth and induced apoptotic cell death by induction of cytosolic and mitochondrial Ca^2+^ overloading and initiation of endoplasmic reticulum (ER) stress signaling pathway, which is mediated by phosphorylation of protein-kinase-like endoplasmic reticulum kinase (PERK) and eukaryotic initiation factor-2α (eIF-2α) and IRE-1, splicing of ER stress-specific X-box transcription factor-1 (XBP-1), cleavage of activating transcription factor-6 (ATF-6), elevation of glucose-regulated protein-78 (GRP-78/BiP) and CCAAT/enhancer-binding protein-homologous protein (CHOP), and cleavage of caspase-12 [54].

**Figure 4:**
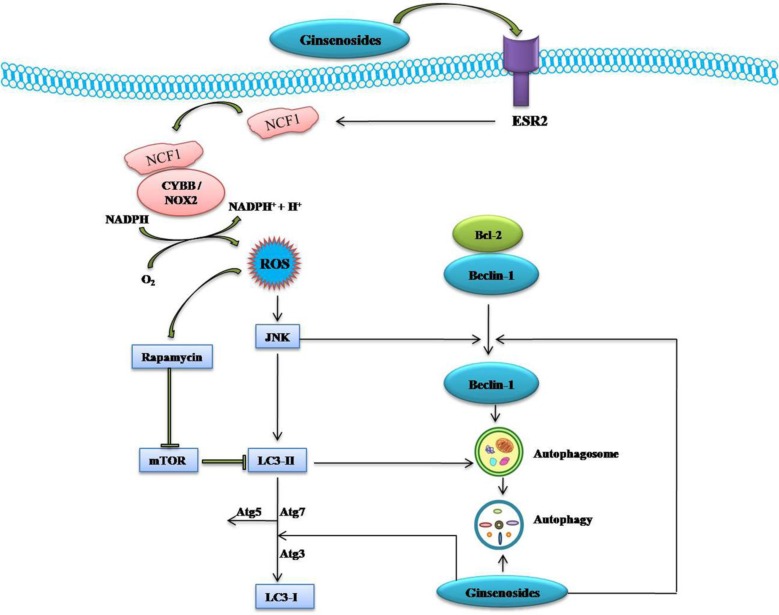
Schematic Diagram of Effects of Ginsenosides on ESR2-NCF1-ROS and ROS-JNK-Autophagy Pathway of Cancer Cells Ginsenoside activate estrogen receptor 2 (ESR2)-neutrophil cytosolic factor 1 (NCF1)-ROS signaling pathway. ROS then activate JNK which in turns phosphorylate Bcl-2 and release Beclin 1 and induce autophagy through up-regulation of ATG5, Atg7, LC3-I and LC3-II. →, Activation; ┤, Inhibition.

Kim et al. [55] demonstrated that compound K (IC_50_=20 μg/ml) inhibited human colon cancer HCT-116 cell growth in a time and dose-dependent manner. Compound K elicited autophagy and apoptosis through the generation of ROS and initiation of JNK pathway association with elevation of autophagy-related protein (Atg, such as Atg5, Atg6, Atg7), microtubule-associated protein 1 light chain 3 (LC3), cleavage of caspase-3 and caspase-9, and modulation of Bcl-2 and Bax protein expression. The pretreated with 50 μM compound K for 24 h enhanced tumor necrosis factor (TNF)-related apoptosis-inducing ligand (TRAIL) induced apoptosis in human colon cancer HCT-116 and HT-29 cells through down-regulated Mcl-1, Bcl-2, survivin, X-linked inhibitor of apoptosis protein (XIAP), and Fas-associated death domain-like IL-1-converting enzyme-inhibitory protein (cFLIP), increased Bax, truncated Bid (tBid), Cyt C, Atg7, LC3-II, and *via* up-regulating autophagy-dependent and autophagy-independent death receptors 5 (DR5) (Figures 3 & 4) [56].

Oral administration of ginsenoside Rg3 (oral administration at 5 or 10 mg/kg, once daily for 15 days) significantly inhibited tumor growth of 87% and promoted the anti-neoplastic efficacy of cisplatin in mice inoculated with CT-26 colon cancer cells. This combination dose-dependently induced apoptosis and down-regulated the expression of Ki-67, VEGF and CD34 in tumor tissues. Ginsenoside Rg3 promoted the efficacy of cisplatin through inhibiting the basal level of nuclear factor erythroid 2-related factor2-(Nrf2) mediated heme oxygenase-1(HO-1)/NAD(P)H quinone oxidoreductase-1(NQO-1) expression in cancer cells [57].

### ROS-mediated anticancer effects of ginsenosides on hepatoma cells

Korean white ginseng extract (KWG) reduced the cell viability in a concentration- and time-dependent manner, induced anti-oxidative effect and stress-induced apoptotic cell signaling, such as JNK and stress-activated protein kinase (SAPK) expressions, release of mitochondrial cytochrome c and activation of caspase-3 in H_2_O_2_-induced HepG2 cells [58]. Fermented black ginseng (FBG) was also showed to defend HepG2 cells against H_2_O_2_-induced oxidative stress by scavenging ROS and increasing both the activity and the expression of cellular antioxidant enzymes, such ascatalase (CAT), glutathione peroxidase (GPx) and manganese-superoxide dismutase (Mn-SOD) via the suppression of MAPK signaling pathways [59]. A recent study showed that ultrasonication processed *Panax* ginseng berry extract (UGB) dose-dependently decreased the cell viability of HepG2 cells with IC_50_ values was 20 μg/mL, and induced apoptotic cell death as associated with Bax activation and Bcl-2 inhibition, increased expression of the cleaved form of caspase-3 and intracellular ROS levels which are responsible for the intrinsic apoptosis pathway [60].

Ginsenosides Rh2 and its octyl ester derivative (Rh2-O) showed concentration- and time-dependent inhibitory effects against the proliferation of HepG2 cells with IC_50_ values of 20.15 and 42.12 μM for Rh2-O and Rh2, respectively. This combination also accelerated apoptosis was associated with disruption of MMP, release of Cyt C, modulation of Bcl-2 family proteins, elevation of caspase-3/-9 activation and PARP cleavage via intracellular ROS generation. Notably, Rh2-O was better antitumor activity than Rh2 plausibly due to its higher cellular uptake [61]. Ginsenosides Rh2 also induces apoptosis in HepG2 cells through the generation of ROS leads to lysosomal membrane permeabilization involving the release of cathepsins B (Cat B), cleavage of Bcl-2 family protein Bid and activation of a caspase-dependent pathway (Figure 3) [62], and through activation of AMP-activated protein kinase (AMPK) *via* ROS generation [63].

Park et al. [64] reported that ginsenosides (GRg3 and GRh2) dose-dependently inhibited the growth of human hepatoma Hep3B cells, and induced apoptosis *via* the generation of intracellular ROS, elevation of Bax/Bcl-2 ratio and initiation of mitochondria-mediated apoptosis pathway (Cyt C and caspase-3). The pretreatment of ginsenosides Rg3 enhanced TRAIL-induced apoptosis in human hepatocellular carcinoma cells *via* CHOP-mediated DR5 up-regulation. The combination of Rg3 (oral dose of 20 mg/kg, daily for 21 days) and TRAIL (intraperitoneal 3 times per week at dose of 3 mg/kg, for 21 days) reduced tumor volume in *in vivo* mouse xenograft model compared with control group along with enhancing TUNEL-positive cells and cleaved caspase-3–positive cells in tumor sections [65].

### ROS-mediated anticancer effects of ginsenosides on breast cancer cells

Compound K dose-and time-dependently inhibited the cell viability of human breast cancer MCF-7 cells with IC_50_ being 35 μg/mL for 48 h. Compound K also accelerated apoptosis MCF-7 cells through intracellular ROS generation as well as down-regulation cyclooxygenase-2 (COX-2) expression and prostaglandin E2 (PGE2) level and the modulation of AMPK signaling [66]. Ginsenoside Rg3 was also proved to induce the apoptosis of human breast cancer MDA-MB-231 cells through up-regulating intracellular ROS generation that was characterized by a down-regulating of Bcl-2/Bax ratio, perturbation of the MTP leading to Cyt C release and initiation of the caspase-3 [67], and inhibiting constitutive activation of NF-κB signaling *via* probably deactivation of ERK and Akt (Protein kinase B) as well as destabilization of mutant p53 through enhancement of Mdm2 binding to mutant p53 protein, which may be responsible for inhibition of Bcl-2 expression [68]. A recent study showed that black Ginseng-silver nanoparticles (BG-AgNps) showed oxidative cell damage and obvious apoptotic activity via generation of ROS and nuclear fragmentation in MCF-7 human breast cancer cells. However, black Ginseng-gold nanoparticles (BG-AuNps) and BG-AgNps revealed non-cytotoxicity in HaCaT and MCF-7 cells [69].

### ROS-mediated anticancer effects of ginsenosides on prostate and cervical cancer cells

A recent study showed that 12-Chloracetyl-PPD concentration-dependently inhibited proliferation and induces G2/M cell cycle arrest and ROS-mediated cell apoptosis in C4-2B prostate cancer cells through down-regulated MDM2 expression, and up-regulated p53 expression [70]. Ginsenoside-Rh2 was showed to induce apoptosis in cervical carcinoma HeLa cells, which is mediated by Ca2^+^ and ROS generation leading to stress-activated protein kinase/extracellular signal-regulated kinase kinase1 (SEK1)/JNK1 initiation and proceeds through the intrinsic pathway that includes Bax-dependent Smac (second mitochondrial activator of caspases) release via mitochondrial depolarization (Figure 3) [71].

### ROS-mediated anticancer effects of ginsenosides on esophageal, gastric, and bladder carcinoma cells

Compound K was showed to dose and time-dependently inhibit the growth and induce the apoptosis of bladder cancer T24 cells through ROS-mediated p38 MAPK pathway via the release of Cyt C, initiation of procaspases-3 and -9, and the alteration of Bax/Bcl-2 proteins ratio [72]. Ginsenoside F2 also induced the apoptosis through accumulation of ROS production followed by a decrease in MTP, Cyt C release, and modulation of apoptosis signal-regulated kinase-1 (ASK-1)/JNK signaling cascade, which triggered the caspase-dependent apoptotic pathway in humor gastric carcinoma SGC7901 cells (Figure 3). Moreover, *in vivo* treatment of ginsenoside F2 (intragastric administration at a dose of 1.6 mg/kg everyday) significantly reduced the tumor growth by 47.14% compared with control group in xenograft mouse model [73]. A recent study showed that ginsenoside Ro inhibits autophagy by hampering with autophagosome-lysosome fusion and lysosomal proteolytic activity by enhancing lysosomal pH and reducing lysosomal cathepsins through the estrogen receptor 2 (ESR2)-neutrophil cytosolic factor 1 (NCF1)-ROS signaling pathway (Figure 4) and thereby sensitized esophageal cancer cells to5-fluorouracil-induced cell death via reduces CHEK1 (checkpoint kinase 1) degradation, increases CHEK1-mediated DNA damage checkpoint arrest [74].

### ROS-mediated anticancer effects of ginsenosides on glioma, glioblastoma, astrocytoma and neuroblastoma cells

It was first recognized that ginsenoside Rh2 inhibited rat C6 glioma cells growth in a dose-dependent manner and accelerated apoptosis mediated by the generation of ROS, DNA fragmentation and the initiation of caspase pathway in a Bcl-XL-independent manner [75]. Ginsenoside Rg1 prevents 1-methyl-4-phenylpyridinium ion (MPP^+^)-induced apoptosis which may be attributed to its antioxidant and anti-apoptotic properties via down-regulating ROS production, activation of JNK and caspase-3 pathway [76]. Ginsenosides CK and Rh2 was showed to accelerate Fas-mediated apoptosis in human astrocytoma CRT-MG cells in a caspase-, mitochondria-, and ROS-dependent manner through distinct apoptotic signaling pathways (Figure 3) [77].

Hwang and Jeong [78] demonstrated that ginsenoside Rb1 prevents 6-hydroxydopamine-induced cell death in human neuroblastoma SH-SY5Y cells through up-regulating heme oxygenase-1 expression, Nrf2 nuclear translocation via anon-genomic, estrogen receptor-mediated Gβ1/PI3K/Akt pathway, thus defending cells from oxidative stress.

The water extract of ginseng (WEG) prevented MPP^+^-induced apoptosis in SH-SY5Y human neuroblastoma cells probably through generation of ROS inhibition and the suppression of mitochondria-dependent apoptotic pathway via up-regulating Bax expression, cytosolic Cyt C and cleaved caspase-3, while down-regulating Bcl-2 expression [79].

Treatment with 20(S)-ginsenoside Rg3 at a ≥10 μM concentration showed a dose-dependent inhibitory effect on U87 human glioma cells proliferation for 3 days, however, chronic treatment at a 20 μM concentration completely inhibited cell proliferation for at least nine days. It also accelerates senescence-like growth arrest via increasing generation of intracellular ROS through Akt initiation and p53/p21-dependent signaling pathways [80]. In another study, treatment of human glioblastoma U87MG cells with 10μM ginsenoside Rg3 for 24 h exhibit a dose-and time-dependent inhibitory effect on their proliferation and elicits apoptosis via up-regulating Bax, while down-regulating Bcl-2 and pro-caspase 3 expression through initiation of ROS by antioxidant enzyme system and MEK signaling pathway [81].

## CONCLUSIONS AND FUTURE PERSPECTIVES

A renewed interest issues in the study of complementary or alternative medicine therapy and less toxic cures for the treatment of different diseases, including cancer. One of the major goals of the treatment of cancers is maximizing efficacy and minimizing adverse effects. Ginsenosides may be a potential complementary or alternative therapy for various cancer patients. We found the potential utility of *Panax* herbs and their derivates, or their active constituents including aglycone such as PPD, 12-chloracetyl-PPD, AD-1, and glycosides such as ginsenoside Rh2, Rg3, F2, Rb1, Rg1, Ro and compound K (Figure 1). In the sheds light of growing literature, ginsenosides possess ample potential for providing diverse mechanisms for treating various cancers (Table 1) that's why it may be tough for cancer cells to become resistance against ginsenosides. Moreover, the properties to kill tumor cells make ginsenosides attractive candidates for drug development. High-throughput expression analysis will help to identify molecular mechanisms and effects of different ginsenosides. The ROS-mediated anticancer effects of different ginsenosides depends on the specific type of various cancer cells involved (Table 1). Some ginsenosides may up-regulate Fas, Bid, tBid, Bax, Bad, p53, p21, p27, p16, Caspase-3, Caspase-8, Caspase-9, ER (endoplasmic reticulum) stress, DR5, CHOP, ATF-6, Smac, PERK, HO-1, EGFR, Beclin 1, Atg7, Atg5, LC3-I, LC3-II, AMP-activated protein kinase, and activate MEK, ASK-1/JNK, ESR2-NCF1-ROS, ER-dependent PI3K/Akt/ Nrf2, P53-CHOP, and ROS-JNK-autophagy signaling pathways. Conversely, these compounds can down-regulate Ki-67, cyclinD1, cyclin E, CDK2/4, cdc25c, cyclin B1, survivin, cFLIP, Mcl-1, XIAP, Bcl-2, cytosol Cat B, Cytosolic vacuolization, CD34, VEGF, COX-2, PGE2, MMP-9 and inhibit thePI3K/Akt signaling pathway (Figure 2). Overall, ginsenosides can inhibit cell proliferation and induce apoptosis, paraptosis or autophagy *in vitro* and *in vivo* by generating ROS in various human cancers. Collectively, these effects suppress cancer cell proliferation via arresting cell cycle progression, inducing cancer cell apoptosis, paraptosis and/or autophagy, and elicit anti-angiogenic and anti-metastatic effects (Figures 3 & 4). Although ROS-mediated effects of ginsenosides exhibit a clear anticancer activity in cancer cell lines, xenograft tumor models or in human clinical trials, it will be further necessary to investigate rigorous multicenter human studies and detailed large scale well-designed cohort clinical trials to confirm the exact effectiveness as an anticancer agents in human patients, thus improving treatment of cancer in near future.

## Abbreviations

CAM: Complementary and alternative medicine
PPD: protopanaxadiol
PPT: protopanaxatriol
ROS: reactive oxygen species
UPB: *Panax ginseng* berry extract
FGB: fermented black ginseng
KWG: Korean white ginseng
BG-AuNps: Black ginseng-gold nanoparticles
BG-AgNps: Black ginseng-silver nanoparticles
S4h: 4 h-steamed American ginseng root extract
WEG: water extract of ginseng
12β: 20(R)-25-methoxyldammarane-3β
25-diol: 12β-O-(L-Chloracetyl)-dammar-20(22)-ene-3β
MAPK: mitogen-activated protein kinases
MDM2: mouse double minute 2 homolog
VEGF: vascular endothelial growth factor
MMP-9: matrix metalloproteinase-9
NOX: nicotinamide adeninedinucleotide phosphate (NADPH) oxidase enzymes
MTP: mitochondrial transmembrane potential
MMP: mitochondrial membrane potential
ER: endoplasmic reticulum
JNK: c-Jun NH2-terminal kinase
mTOR: mammalian target of rapamycin
TRAIL: tumor necrosis factor (TNF)-related apoptosis-inducing ligand
XIAP: X-linked inhibitor of apoptosis protein
cFLIP: Fas-associated death domain-like IL-1-converting enzyme-inhibitory protein
tBid: truncated Bid
CHOP: C/EBP homologous protein
DR5: death receptors 5
Nrf2: nuclear factor erythroid 2-related factor 2
HO-1: heme oxygenase-1
NQO-1: NAD(P)H quinone oxidoreductase-1
Cat B: cathepsins B
AMPK: AMP-activated protein kinase
COX-2: cyclooxygenase-2
PGE2: prostaglandin E2
ERK: extracellular signal-regulated kinase
Akt: protein kinase B
SEK1: stress-activated protein kinase/extracellular signal-regulated kinase kinase1
Smac: second mitochondrial activator of caspases
ASK-1: apoptosis signal-regulated kinase-1
ESR2: estrogen receptor 2
NCF1: neutrophil cytosolic factor 1
CHEK1: checkpoint kinase 1
ATF6: Activating transcription factor 6
PARP: poly (ADP-ribose) polymerase
EGFR: epidermal growth factor receptor
Atg: autophagy-related protein
Beclin-1: autophagy-related protein 6
LC3: microtubule-associated protein 1 light chain 3
FADD: Fas-associated death domain
PERK: PKR-like endoplasmic reticulum kinase
ROS: Reactive oxygen species
CK: compound K
PI3K: phosphatidylinositol 3-kinase
